# The diagnostic dilemma in a patient with neuroleptic malignant syndrome during the COVID‐19 pandemic: A significant increase in acute phase reactants

**DOI:** 10.1002/ccr3.7734

**Published:** 2023-08-02

**Authors:** Forouzan Elyasi, Mehran Zarghami, Arghavan Fariborzifar, Hamed Cheraghmakani, Mahboobeh Shirzad, Feteme Kazempour

**Affiliations:** ^1^ Sexual and Reproductive Health Research Center, Psychiatry and Behavioral Sciences Research Center Addiction Institute, Mazandaran University of Medical Sciences Sari Iran; ^2^ Department of Psychiatry, Faculty of Medicine Mazandaran University of Medical Sciences Sari Iran; ^3^ Psychiatry and Behavioral Sciences Research Center Addiction Institute, Mazandaran University of Medical Sciences Sari Iran; ^4^ Mental Health Research Center, Psychosocial Health Research Institute (PHRI), Department of Psychiatry, School of Medicine Iran University of Medical Sciences Tehran Iran; ^5^ Neurology Department, Faculty of Medicine Mazandaran University of Medical Sciences Sari Iran; ^6^ Department of internal Medicine, Faculty of Medicine Mazandaran University of Medical Sciences Sari Iran; ^7^ Student Research Committee, Faculty of Medicine Mazandaran University of Medical Sciences Sari Iran

**Keywords:** acute phase reactants, antipsychotic, neuroleptic malignant syndrome, side effect

## Abstract

**Key Clinical Message:**

In some patients, neuroleptic malignant syndrome is accompanied significant high levels of erythrocyte sedimentation rate (ESR), C‐reactive protein (CRP).

**Abstract:**

Neuroleptic malignant syndrome (NMS) is an idiosyncratic life‐threatening adverse reaction and usually triggered in response to antipsychotic drugs. In addition, leukocytosis and increased muscle enzymes levels (especially creatine phosphokinase) are observed in NMS. In addition, a transient increase in different types of acute phase reactants in NMS has been mentioned. This article describes a woman treated with haloperidol, perphenazine, escitalopram, and alprazolam because she developed catatonic symptoms after psychological stress. She suffered from NMS symptoms and had elevated CRP and ESR levels, among other signs and symptoms. Given the COVID‐19 pandemic and reports of co‐occurrence of catatonia and NMS and COVID‐19 and elevated erythrocyte sedimentation rate (ESR) and C‐reactive protein (CRP), this patient was a diagnostic dilemma. After consultation with the consultation‐liaison psychiatry units, she was managed adequately with electroconvulsive therapy and lorazepam.

## Abstract 2

Erythrocyte sedimentation rate and C‐Reactive Protein (CRP) Test in different days.

## 1 INTRODUCTION

Neuroleptic malignant syndrome (NMS), as an acute, rare, life‐threatening, and idiosyncratic condition, is generally triggered in reaction to dopamine receptor antagonist agents, such as typical or conventional antipsychotics, atypical antipsychotic agents, and metoclopramide, levodopa, and amantadine withdrawal.^1^ This complication is characterized by very high fever, muscle rigidity, tremors, autonomic nervous system (ANS) instability, hyperhidrosis, dysphagia, altered level of consciousness (ALOC), leukocytosis, and elevated creatine phosphokinase (CPK).^2^ The underlying pathophysiological mechanisms of NMS are still vague,^3^ and those caused by antipsychotics are often associated with its impact on dopamine in the mesocortical‐nigrostriatal pathways and the hypothalamic nuclei.^3^ As shown by previous studies, the incidence rate of NMS has had a downward trend over the recent years, due to the growth in the prescription of atypical antipsychotic drugs compared to the typical ones, reaching 0.01%–0.02% in cases undergoing treatment, and even 0.07%–3.22% in some reports.^1^, ^2^, ^3^, ^4^ On the contrary, malnutrition, dehydration, iron deficiency, exposure to extreme heat, hyponatremia, and thyrotoxicosis have been characterized to increase the risk of this condition.^5^ The mortality rate caused by NMS had been already estimated to be over 30%. However, this value has recently dropped and reached about 10% due to the increase of awareness among physicians and the administration of atypical antipsychotics or second‐generation antipsychotics.^5^ Of course, proper treatment requires an accurate diagnosis, and in this way, there are several important differential diagnoses that complicate the diagnosis.

In this context, catatonia is mentioned, which refers to a group of symptoms, including impaired communication, unusual or lack of movement, and some behavioral problems, attributable to psychiatric or neuromedical causes. In addition, catatonia have been associated with structural brain defects, neurological disorders (e.g., epilepsy, toxic‐metabolic diseases, and infections), a number of systemic disorders affecting the brain, and mental disorders in their idiopathic forms (such as, affective psychosis and schizophrenia).^6^ Catatonia has been described as one of several neuropsychiatric manifestations associated with COVID‐19 infection.^7^, ^8^ Detailed descriptions of catatonic symptoms in COVID‐19 disease are largely lacking in the literature. These reports also suggest an inflammatory basis for COVID‐19‐related catatonia. In the current pandemic, an association between the development of catatonic symptoms in COVID‐19 patients and the concordant rise in serum pro‐inflammatory markers has been showed for the first time. In general, there is also a co‐occurrence of catatonic symptoms with nonviral inflammatory or autoimmune conditions, and with biochemical inflammatory markers in the neuroleptic malignant syndrome.^7^, ^8^, ^9^, ^10^

Since antipsychotic drugs are a potential cause of NMS, this complication has recently been introduced as a severe form of neuroleptic‐induced catatonia.^11^

Autoimmune encephalitis (AE) also has some neuropsychiatric manifestations, and is in the differential diagnosis with NMS, based on high fever, the symptoms of catatonia, and movement disorders.^11^ The *N*‐methyl‐d‐aspartate receptor encephalitis is the most common form of AE, which appears in young adults, mainly in women. This syndrome often begins in adults with prodromal viral symptoms, and then progresses to mental or behavioral disturbances and insomnia, accompanied by ALOC, seizures, unusual movements, and autonomic dysfunction and instability that can lead to central hypoventilation syndromes.^12^

Some characteristic laboratory abnormalities of NMS are the changes in acute phase reactants (APR)s. APRs are non‐specific inflammatory markers that increase or decrease markedly during inflammation.^13^ Leukocytosis and elevated muscle enzymes (particularly CPK) are the APRs observed NMS.^14^ This increase in different APRs types in NMS has been reported in many studies.^10^ Various mechanisms of the induction of the acute phase response in NMS are autoantibody production, virus–drug interaction, heat stress, muscle break‐ down and psychological stress.^15^

However, there is an upward trend in CPK, erythrocyte sedimentation rate (ESR), C‐reactive protein (CRP), interleukin‐6 (IL‐6), alpha‐1 antichymotrypsin (ACT), and fibrinogen, the levels of iron (Fe) and albumin levels(AL) decrease. Such responses correspond to the APR activation.^15^ The positive values of APR, ESR, CRP, IL‐6, ACT, and fibrinogen peaked after 72 h. In NMS, serum levels of negative APRs, such as serum AL and Fe are low, but gradually redouble and reach the normal range, along the lines of clinical improvement. This transient APR profile is consistent with the changes in temperature, creatine kinase (CK) levels and the clinical course of the syndrome, suggesting that APR may directly contribute to the pathophysiology of NMS.^15^ A sudden drop in Fe levels is regularly observed in connection with NMS, which is a key manifestation of APR.^16^ As mentioned in some publications, APR is considered less important than CPK,^11^ although APR may be involved in the pathogenesis of NMS, suggesting a possible role in disease initiation.^17^ In this case report, a middle‐aged catatonic woman, developed NMS symptoms along with elevated CRP and ESR levels due to typical antipsychotics.

## 2 CASE REPORT

**FIGURE 1:**
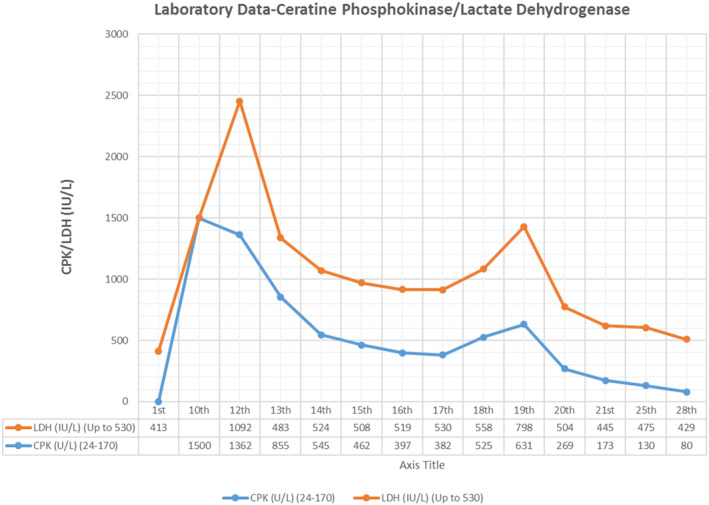
Creatine phosphokinase and lactate dehydrogenase levels in different days.

The patient was a 61‐year‐old married woman who suffered from multiple and short‐term attacks of mutism and spasms in the hands, the day after experiencing a stressor when confronted with a thief stealing from their neighbor. Then she hospitalized in a general hospital in 2022. Based evaluation by a psychiatrist, the patient noted mutism, marked negativity, and resistance to recommendations, prescriptions and movements. Therefore, the patient was diagnosed with catatonia, and the possibly brief psychotic disorder (BPD). Neurological consultation, an electroencephalogram (EEG), brain computed tomography (CT) scan, and various paraclinical examinations were performed, but no pathological changes were detected Therefore, the patient received an intravenous (IV) dose of diazepam 10 mg in the evening and alprazolam 0.5 mg orally twice daily; and escitalopram 10 mg at noon. On the second day of hospitalization, haloperidol tablets 0.5 mg twice a day were added to her medication regimen, but she was then prescribed a haloperidol 5 mg dose with biperiden 5 mg intramuscularly due to her restlessness and persistent symptoms and refusal to take oral medications. On the third day, patient obeyed but sometimes screamed in the ward. On the fourth day of admission, relative verbal communication, negativism, and the spontaneous movements of the body parts were observed, oral haloperidol and injectable diazepam were discontinued and started on perphenazine 4 mg twice a day. Electroconvulsive therapy (ECT) was refused by the anesthesia services and was not carried out despite her psychiatric plans. Because the infectious disease specialist and neurologist were suspected to herpes simplex virus, magnetic resonance imaging (MRI) of the brain and cerebrospinal fluid (CSF) analysis were performed by real‐time polymerase chain reaction (PCR), which were reported negative. On the sixth day, she was again injected with haloperidol injection due to severe agitation. On Day 7 of hospitalization, the dose of perphenazine was increased to 8 mg twice daily. By Day 10, the patient was partially compliant, but could not swallow solid food. Perphenazine was stopped due to elevated CPK levels (1500 U/L) and alprazolam 0.5 three times a day and escitalopram continued (Figure 1). On the 12th day, the patient with the possible diagnosis of NMS was transferred to a psychiatric hospital and then to the psychosomatic medicine ward in a general teaching hospital in Sari‐Iran.

During her stay in the psychosomatic medicine ward, the patient presented with recurrent symptoms of catatonia, including echolalia, echopraxia, negativism, posturing, verbal stereotypes (ask for permission) and muscle rigidity. She was presented with excitement, staring, echolalia, echopraxia, muscle rigidity, negativism, and gegenhalten, based on the results of the Modified Bush‐Francis Catatonia Rating Scale.^11^ Other symptoms include tremors, mutism, and excessive sweating. The patient also met the Levenson criteria for the diagnosis of NMS.^18^

**FIGURE 2:**
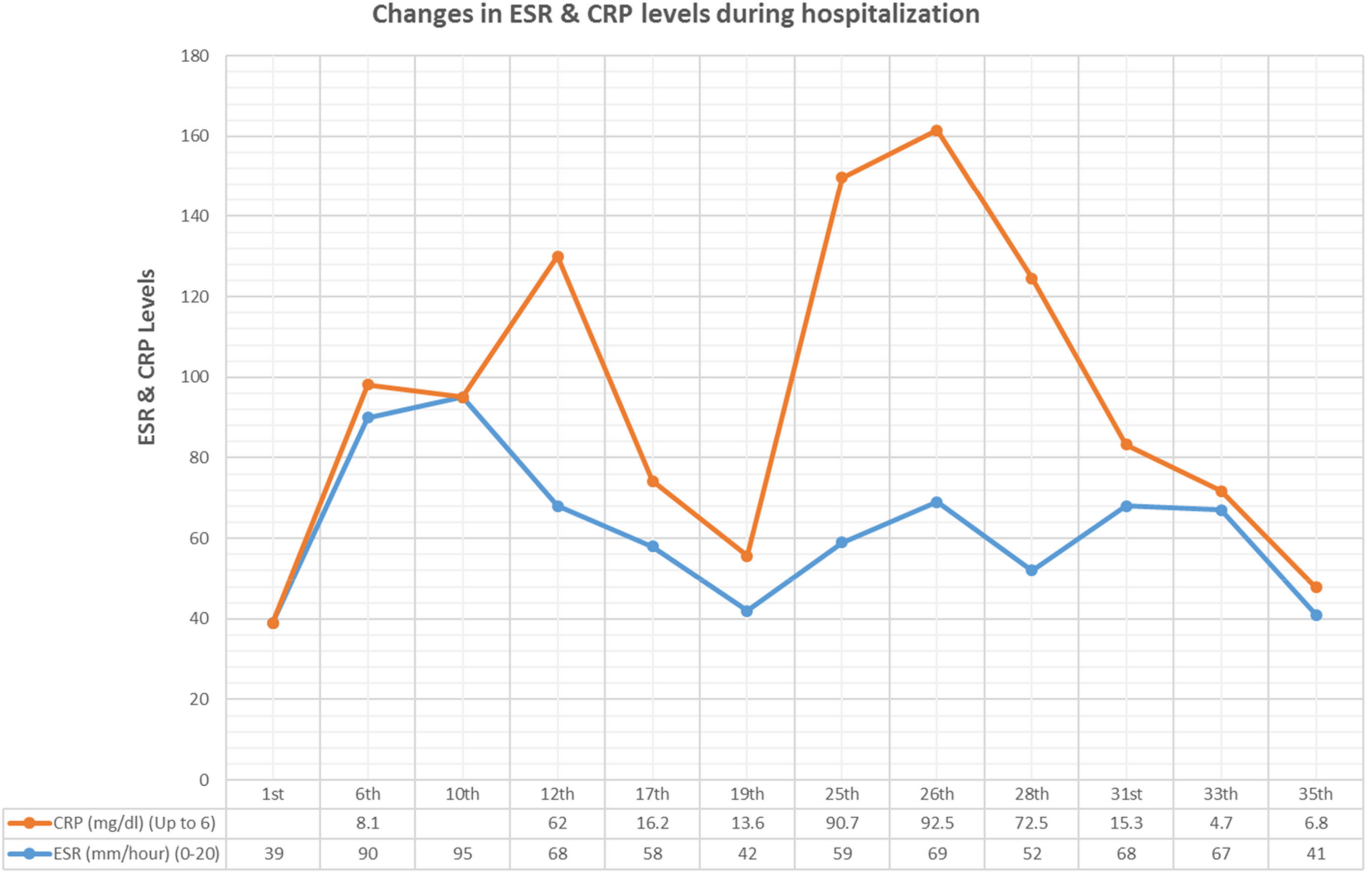
Erythrocyte sedimentation rate and C‐reactive protein (CRP) test in different days.

**FIGURE 3:**
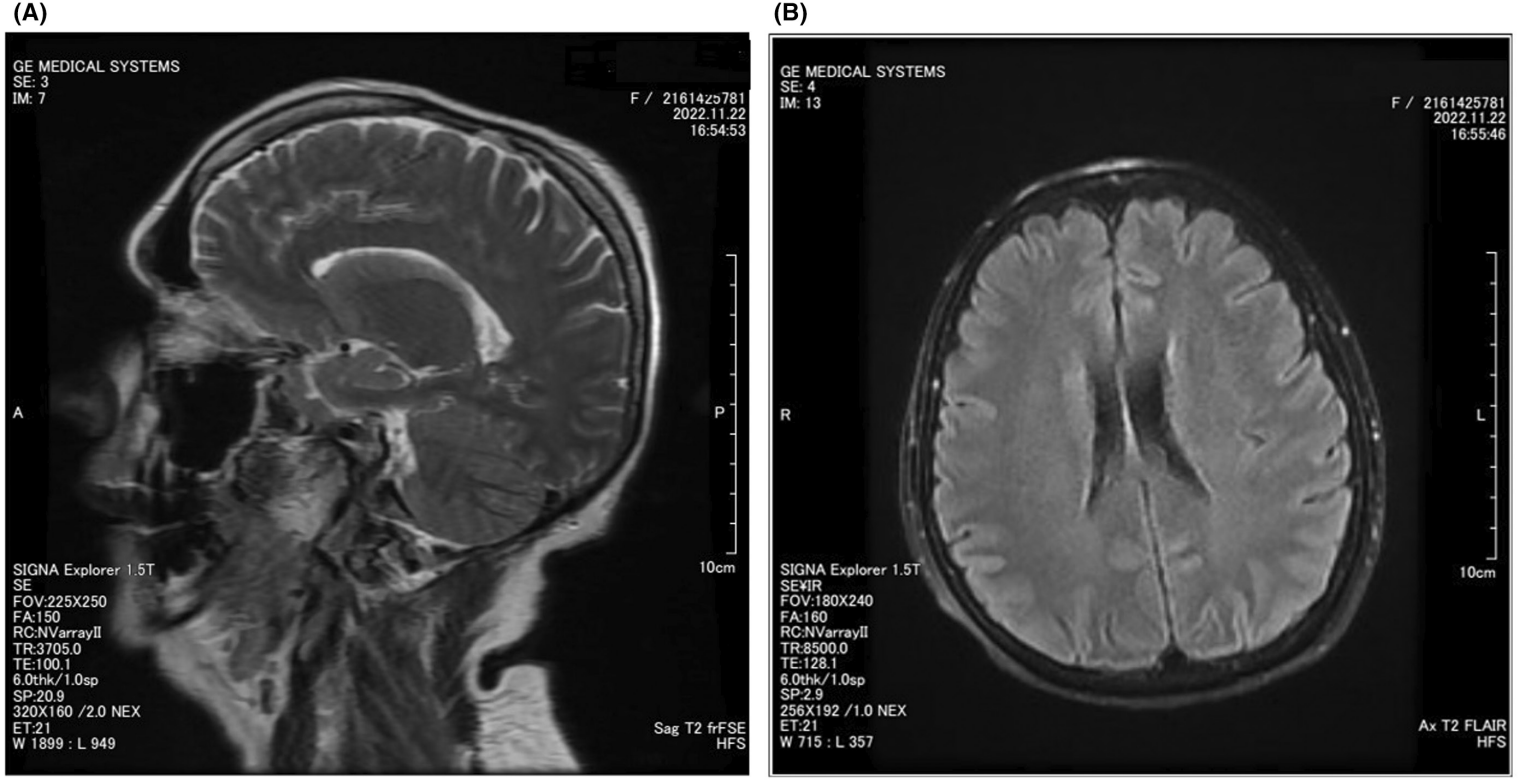
(A, B) Patient's brain MRI.

**FIGURE 4:**
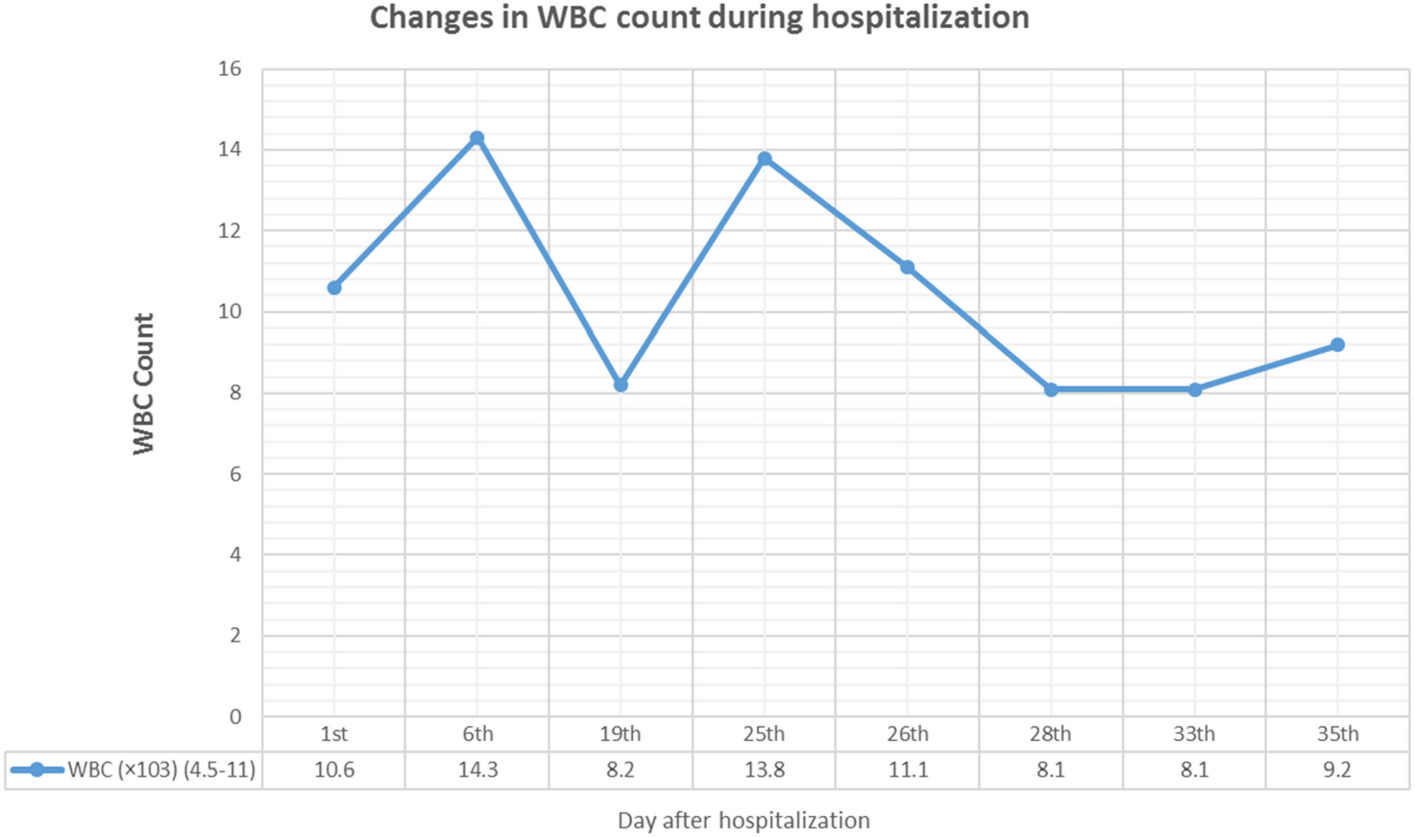
Patient's white blood cell count.

**FIGURE 5:**
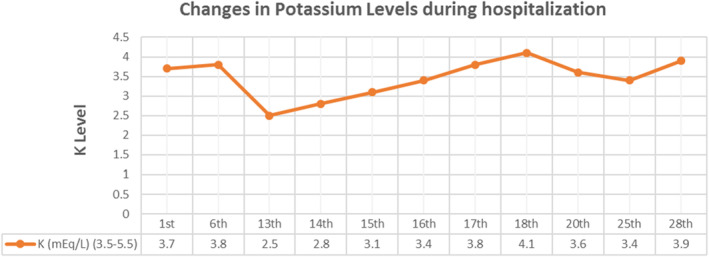
Potassium levels in different days.

Due to the high ESR and CRP levels (Figure 2), as well as the presence of symptoms, a grand round was organized with the participation of a neurologist, a subspecialist of the intensive care unit, an infectious disease specialist, three psychiatrists, and a pulmonologist and then a CSF analysis and a MRI of the brain were performed once again in order to rule out the neuromedical causes particularly AE, and coronavirus disease 2019 (COVID‐19) (Figure 3A,B). Differential diagnosis of NMS, including Serotonin Syndrome, Malignant Hyperthermia, Central Nervous System (CNS) infections, AE and other similar conditions were further considered but ruled out (Figures 4 and 5). To test for CNS infections, the patient underwent a lumbar puncture again and her CSF was analyzed for infection factors and autoantibodies, but the tests were negative. The results of the CSF analysis are presented in Table 1.

**TABLE 1:** Cerebrospinal fluid (CSF) analysis.

Item	Normal Range	Result
CSF Alb	3.5–5.2 (mg/dL)	1.1 (mg/dL)
CSF Protein	15–40 (mg/dL)	44 (mg/dL)
CSF LDH	225–480 (IU/L)	75 (IU/L)
CSF Glucose	70% of blood sugar	64 (mg/dL)

Since November 21, 2022, on the first day of hospitalization in the psychosomatic medicine ward, an injection of lorazepam 1 mg intramuscularly three times a day prescribed, and from the fifth day ECT was started for eight sessions. After the first ECT session, her speech incoherence decreased. So she could correctly call the interviewer “Doctor”, but she addressed her by the name of her first‐grade teacher. After the second ECT session, her speech improved significantly, but she had visual hallucinations. She was always talking about her pregnancy, her previous births and the baby she lost a long time ago. The patient was also feeling persecution toward some nurses, and even felt frightened when they were in her room. On the 14th day of admission to the psychosomatic ward, the dose of lorazepam was decreased to 2 mg/day, but the patient became febrile for 5 h and her CRP increased again from 13.6 to 90.7 mg/dL(Figure 2). After the lorazepam dose was increased back to the previous dose, CRP began to decrease.

During hospitalization, the NMS symptoms were first subsided, CPK and lactate dehydrogenase (LDH) levels decreased (Figure 1), the symptoms of catatonic symptoms were subsided, then psychotic symptoms appeared and delirium symptoms became gradually noticeable. Therefore, the dose of lorazepam was gradually reduced, and then replaced with oral lorazepam. Finally, after 27 days of hospitalization at the second hospital (i.e., 39 days after symptom onset), the patient was discharged on ferrous sulfate and oral lorazepam (3 mg/day). At that time, complete examinations of the rheumatology laboratory tests and mammography were carried out to rule out possible paraneoplastic syndromes and rheumatic diseases which came out negative. Over a period of 2 months and during outpatient visits after discharge from the hospital, she had no psychotic symptoms or mood disorders, and functioned normally, so the dose of lorazepam reached to 1 mg at night. In the fifth month after discharge, lorazepam was stopped and she was normal.

## 3 DISCUSSION

In this case, a middle‐age woman with catatonia had NMS symptoms as well as elevated CRP and ESR levels. The patient has good job performance and good interpersonal relationships throughout her life; although she had a history of an 18‐h brief psychotic episode about 20 years ago, and the current symptoms had further occurred following a psychological stressor. Because the patient's catatonic symptoms began acutely, it was extremely important to rule out neuromedical causes. In view of the COVID‐19 pandemic and the reports of co‐occurrence of NMS and COVID‐19^19^ and elevated ESR and CRP, this patient also underwent a PCR test for COVID‐19 which was negative. The examination for paraneoplastic syndromes and rheumatic diseases was also negative. This patient had serum iron, total iron‐binding capacity, and ferritin levels of equal to 51 μg/dL, 211 μg/dL, and 232 ng/mL respectively, indicating iron deficiency. On this point, Anglin et al. had measured serum iron levels in 33 patients with NMS. The score was low in 32 out of 33 cases, with an average of 5.38 ± 2.78 mmol/L (compared to the normal range of 13–32). In all patients, the serum iron level had further returned to the normal range following the improvement of NMS. In the cases whose serum iron level had been at the lowest point, it had returned to the highest level after recovery.^15^ This could be strong evidence that iron was essential for the normal function of dopamine D_2_ receptors.^20^ The decrease in serum iron levels in APR could therefore be the result of a decrease in functional dopamine D_2_ receptors in the brain.^15^

Accordingly, several mechanisms have been accordingly proposed to induce APR in NMS, including the autoantibody production, virus–drug interactions, heat stress, muscle breakdown, and psychological stress.^15^ APR in NMS is the characteristic of neutral innate immune system. Other evidence also suggests that the activation of the adaptive immune responses is often involved.^15^ Patients with a history of NMS thus have autoantibodies against neurotransmitter receptors that are four standard deviations higher than in healthy controls.^21^ Before developing NMS, patients often are agitated and psychotic, are subject to certain psychological stresses that trigger APR.^22^ Another potent factor is probably catatonia, which immediately precedes NMS, and is associated with very high fever.^15^ In this regard, animal studies have shown that acute stress, such as immobilization and inevitable shock, increases IL‐1‐β levels in the brain, suggesting a centrally activated APR in response to stress. This has been further pointed out in recent decades that viruses can interact with drugs, and lead to adverse drug reactions. From this point of view, viral infections can impair immune regulation and reduce drug tolerance. In this sense, Anglin et al. have noted that NMS may be associated with a drug‐virus interaction; such that a predisposing viral disease can result in a severe APR for antipsychotics.^15^

As well, Rosebush et al. in a case report on NMS and APR, had introduced a healthy, non‐smoker 17‐year‐old boy, who was hospitalized with a diagnosis of possible NMS. He was treated with intramuscular zuclopenthixol acetate 100 mg for 9 days for catatonia, aversive behavior and psychosis. The next day, the dose was increased to 200 mg, and 7 days after taking it, he developed a fever, became rigid, and unresponsive. Based on the laboratory test results, CPK and aspartate aminotransferase were 4329 and 159 U/L respectively. The APR was also high. Positive APR values such as ACT, fibrinogen, ESR, and IL‐6 again peaked at 72 h and returned to the normal range within 14 days. In addition, CPK peaked on Day 4, and returned to the normal level on Day 14. On the other hand, the negative APR, such as serum iron and AL levels were low during the acute phase of the disease, and gradually increased with clinical recovery. The serum levels of CRP‐1, 2, 3, 4, and 6 levels were equal to 27, 30, 40, 1.53, and 49.7 mg/L respectively, and had reached less than 5 by Day 14. The highest serum IL‐6 concentration was equal to 187 pg/mL on Day 1.^17^

Soh et al. had also reported two cases of NMS with co‐occurrence of COVID‐19. One of the patients was a 44‐year‐old man with acute respiratory distress syndrome caused by COVID‐19, who was treated with favipiravir and then risperidone on Day 5 due to delirium. On the seventh day, his temperature had reached to 40.8°C and the CPK had elevated. The patient also had tachycardia, tachypnea, ALOC, and excessive sweating, So NMS had been diagnosed, and both favipiravir and risperidone discontinued. The underlying mechanism of NMS in COVID‐19 patients treated with favipiravir remains unknown.^19^

Lethal catatonia (LC) is one of the most important differential diagnoses of NMS. Also known as malignant catatonia, LC is a dangerous condition that with regards to a neuropsychiatric, toxic‐metabolic, or general medical disease and includes catatonic symptoms and ANS dysregulation and has co‐occurs with NMS.^23^, ^24^ Some experts believe that both NMS and LC have similar clinical features, as well as a single biochemical pathway of reduced GABA_A_ inhibition in the frontal corticostriatal tracts, and therefore both respond to a combination of benzodiazepines and ECT.^23^, ^25^, ^26^ Benzodiazepines improve both conditions by increasing GABA activity. In addition, ECT can cause a neural storm with increased GABA transmission.^7^, ^27^ In terms of differential diagnosis, LC presents with catatonic symptoms, bizarre behavior and psychiatric disturbances. Conversely, NMS traditionally has been associated with antipsychotic drug use, with acute onset of fever, stupor, extrapyramidal symptoms and ANS dysfunction.^23^, ^27^ Therefore, the diagnosis of LC would be apparent if the history does not reveal any exposure to the neuroleptic drugs.^23^, ^28^

## 4 CONCLUSION

Since morbidity and mortality increase when treatment is delayed or subtherapeutic with using ineffective and less aggressive techniques,^16^ careful consideration of the evolution of NMS in the management of patients with APR, particularly during COVID‐19 pandemic is essential and necessary. APR may be involved in the NMS pathogenesis, which suggests its potential illness‐inducing role. At the point when clinical and paraclinical features suggest NMS, early consultation with consultation‐liaison psychiatry unit and collaborative management is strongly recommended.

## AUTHOR CONTRIBUTIONS

**Forouzan Elyasi:** Conceptualization; investigation; project administration; supervision; writing – original draft; writing – review and editing. **Mehran Zarghami:** Methodology; supervision; writing – review and editing. **Arghavan Fariborzifar:** Data curation; writing – review and editing. **Hamed Cheraghmakani:** Investigation; writing – review and editing. **Mahboobeh Shirzad:** Writing – review and editing. **Feteme Kazempour:** Investigation.

## CONFLICT OF INTEREST STATEMENT

The authors declared no competing interests in this study.

## ETHICS STATEMENT

The research proposal was approved by the Ethics Committee affiliated with Mazandaran University of Medical Sciences [IR. MAZUMS.REC.1402.139].

## CONSENT

Written informed consent was obtained from the patient to publish this report in accordance with the journal's patient consent policy.

## Data Availability

This is a case report of a single patient. All data provided during this study are included in this article.
